# “It’s Just Always Eating”: The Experiences of Young People Growing up Medium Chain Acyl-coA Dehydrogenase Deficiency

**DOI:** 10.1177/23333936211032203

**Published:** 2021-08-16

**Authors:** Hilary Piercy, Charlotte Nutting, Sufin Yap

**Affiliations:** 1 Sheffield Hallam University, UK; 2 Sheffield Children’s Hospital NHS Foundation Trust, UK

**Keywords:** qualitative, young people, metabolic disorder, medium chain acyl-CoA dehydrogenase deficiency, MCADD, England

## Abstract

Medium chain acyl-CoA dehydrogenase deficiency (MCADD) is a rare metabolic disorder, and commonly now part of newborn screening programs. Those diagnosed at birth are now progressing from childhood to adulthood. The study aim was to explore young people’s experiences of living with MCADD and managing their condition. A descriptive qualitative study design involving semi-structured interviews with 12 participants aged 10 to 15 years, recruited from one regional pediatric metabolic disorder service in England. Data were analyzed using thematic analysis. The two major themes were “Eating for energy” and “Growing into a self-management role.” Self-monitoring and self-management skills had been nurtured from early childhood by parents and healthcare providers. Young people’s anxieties concerned having to maintain adequate energy input to stay safe and the associated burden of responsibility. Growing up with MCADD presents specific challenges. Self-management and ongoing support are important for dealing with those challenges.

## Background

Medium chain acyl-CoA dehydrogenase deficiency (MCADD) is the most common inherited disorder of fatty acid oxidation. The incidence is highest in populations of Northern Europe and affects between 1 in 9,000 to 1 in 10,000 newborns in the United Kingdom (Grosse et al., 2006; Oerton et al., 2011). In this rare metabolic condition, medium chain fatty acids are improperly metabolized and accumulate in the blood which can lead to metabolic crisis which presents clinically as hypoglycemia and lethargy. Without rapid treatment, this can result in liver damage, brain damage, and death (Leonard & Dezateux, 2009).

Over the past decade, screening for MCADD has been embedded into newborn screening programs in the United States of America, Canada, and many European countries including the UK (Jameson & Walter, 2019; Therrell et al., 2015). This has resulted in improved health outcomes because detecting the condition and instigating management in the first few days of life has significantly reduced the likelihood of adverse events including death (Lindner et al., 2011; Nennstiel-Ratzel et al., 2005; Oerton et al., 2010; Wilcken et al., 2009). MCADD is managed by diet, ensuring that food intake is sufficient to meet metabolic demands and avoiding prolonged periods of fasting. For children over 1 year old this involves eating regularly and maintaining a “safe fasting interval” of 12 hours for life when well. (British Inherited Metabolic Diseases Group, 2016). During periods of illness, metabolic requirements increase with greater likelihood of metabolic crisis. This necessitates specific management involving an emergency dietary/feeding regime of glucose polymer feeds every 2 to 3 hours with age-appropriate carbohydrate content (British Inherited Metabolic Diseases Group, 2016).

Our understanding of what it means to grow up with MCADD is currently limited to parental perspectives and primarily concerned with understanding the challenges associated with infancy and early childhood. Initial challenges associated with managing dietary regimes subside as parents become more confident in their abilities. However, parents report ongoing challenges associated with involving others and ensuring that they can be safely entrusted with the care of their child (Khangura et al., 2016; Piercy et al., 2017; Siddiq et al., 2016).

Those first detected with MCADD through newborn screening programs are progressing from childhood to adulthood and transitioning from pediatric to adult healthcare services, an event that is widely recognized as problematic and associated with poorer outcomes for many health conditions (Chu et al., 2015; van Staa et al., 2011). “Young people” is a non-prescriptive term used to describe this population in contrast to the terms “adolescent” and “teenager” which carry largely negative connotations (Griffin, 2013). The term “young people” is widely preferred by health and social care services in the UK and is used throughout this paper.

Insights into the experiences of those growing up with MCADD are important for informing the transition process and wider healthcare provision to enable care to be tailored and outcomes maximized for this group of people. No work has been conducted in this area to date. The aim of this study was to explore young people’s experiences of living with MCADD and managing their condition.

## Methods

We used a descriptive qualitative study design, as detailed by Sandelowski (2010) aligned to a social constructionism standpoint that understands meanings as constructed by human beings as they engage with the world that they are interpreting (Burr, 2006). Participants were recruited from one Regional Pediatric National Health Service (NHS) Specialized Metabolic Disorder Service in England. The service cares for individuals from birth to their 16th birthday when they transfer to adult services. All those registered with the service and aged 10 to 15 years were eligible to participate. The metabolic consultant provided verbal and written information about the study to prospective participants and their parent as part of routine care. A member of the research team then phoned the parent for recruitment purposes.

Our recruitment approach was informed by consultation with a research advisory group composed of young people who also contributed to development of recruitment materials. We used a single study information sheet that was designed for the young person with the aim of encouraging a family discussion about the project and ensuring that the young person as well as their parent fully understood the project and was willing to be involved. We obtained written informed consent from the parent and written informed assent from the young person prior to data collection.

Data collection involved a single semi-structured interview which was conducted either at the participants’ home or on university premises, and with a parent absent or present according to the participants’ wishes. The interview schedule comprised a small number of key questions and probes to enable in-depth discussions that adequately captured individual experiences and perceptions (Cresswell, 2008). Interviews were digitally recorded and transcribed verbatim. They lasted from 20 to 50 minutes. At the end of the interview, participants were invited to choose their own pseudonym for reporting purposes. All participants received a gratuity of a £15 shopping voucher for contributing to the study.

Data were analyzed using a thematic approach (Braun & Clarke, 2006) and supported by QUIRKOS, a qualitative data analysis software program. This involved familiarization with the entire dataset, inductive coding of all interviews and development of a thematic structure with overarching main themes and sub themes. All members of the research team contributed to the analytic processes. Authors 1 and 2 independently coded all interviews and then compared these to agree an overall analytic structure of themes and sub themes. This was subsequently revised and finalized through iterative analytic processes and discussions with the rest of the research team. We received ethical approval from the London-Chelsea Research Ethics Committee (reference 19/LO/0552) and research governance approval from the Health Research Authority.

## Findings

We recruited 12 participants from a total cohort of 14. In the other two cases, one parent and one young person declined to participate. Table 1 provides an overview of participants.

**Table 1.** Details of Study Participants.

Pseudonym	Sex	Age (years)
Jasmin	Female	10
George	Male	11
Nadia	Female	11
Ally	Female	11
Simon	Male	11
Noah	Male	11
Charmaine	Female	12
Frankie	Female	13
Amber	Female	13
Sarah	Female	14
Mia	Female	14
Marie	Female	15

The two major themes identified from the data were “Eating for energy” and “Growing into a self-management role.” “Eating for energy” contained the two sub themes of “Maintaining a regular energy supply” and “Keeping the energy levels topped up.” “Growing into a self-management role” contained the two sub themes of “Understanding my condition” and “Practical steps to independent management.”

### Theme 1. Eating for Energy

Eating for energy focuses on how the dietary requirements associated with day-to-day management of MCADD translated into the lives of the young people and the associated challenges they identified. “Maintaining a regular energy supply” examines their overall understanding of and approach to dietary management. “Keeping the energy levels topped up” explores what was required to enable them to engage in normal day to day activities and the social aspects of managing those requirements.

## Maintaining a Regular Energy Supply

All the young people understood the importance of regular food intake to keep them safe and well. Charmaine described eating and drinking as *“her medicine”* and 11 year-old Ally summarized the situation when she said *“I have to eat regularly.”* Most offered more detailed information, explaining the importance of eating regularly and not missing meals, particularly breakfast which plays a key role in protecting the safe fasting interval, and their specific dietary requirements. Sarah explained that *“The diet is supposed to be high carb and low fat . . . we’ve got to make sure we get the right stuff . . . we’ve got to have a balanced diet.”* Marie explained that *“It’s just like in a routine now. I know I’ve got to eat in a morning and make sure I eat at school and after school and stuff. Just make sure I eat regular and stuff, that’s about it.”*

Some of the girls reported that they found the need to eat difficult, and the possible consequences of not doing so, was an ever-present threat. Charmaine explained that “*You don’t always want to eat”* and Mia expressed similar feelings when she said *“I do try and eat when I can but sometimes I’ve just ate too much and I can’t eat anymore.”* This was a particular problem when they were unwell as Nadia explained: *“If I actually do have some kind of bug, they just tell me to eat when that’s what I might need the least.”*

Several young people made specific reference to the consequences of not eating that indicated ongoing anxieties about their condition. Marie said “*most of the time, I don’t really want to eat but I know I’ve got to else I’ll be in hospital*.” In some cases, this translated into concerns about keeping themselves safe. Simon asked “*How can I keep reminding myself to eat*?” and Mia expressed similar concerns when she explained “*I just always worry that what if I don’t eat while I’m out*.”

## Keeping up the Energy Levels

A recurring feature was the way the young people conceptualized their bodies as a fuel tank, constantly emptying and needing to be topped up. Simon could *“feel my energy going down”* and Frankie explained that *“If my levels go down it could make me ill.”* Sugary drinks and snacks provided the energy top-ups they needed to undertake normal daily activities. Charmaine explained *“I need to keep sugar in my system, I need to have snacks in between to keep me boosted up and all right.”* Mia highlighted the need to top up when exercising, explaining that *“When I’m doing physical activities I have to drink a sugary drink and I have to keep eating a lot.”* Nadia made a similar point, linking it to the extra energy demands of exercise: *“. . .in PE especially, because that’s when I need a lot more energy for it. I usually have a snack before I do PE.”*

Access to top ups during the school day was managed in different ways and created variable challenges for the young people. In primary school, class teachers all knew about their MCADD, provided some degree of monitoring and ensured they had ready access to their snacks in the classroom.

Different processes operated in secondary schools and the change of school had been a source of anxiety for some. Older participants explained the processes that operated in their school. Charmaine described a managed system whereby a member of staff was responsible for ensuring she had snacks at regular intervals during the day and several including Noah reported a pass system that enabled them to leave lessons if needed and gave them access to medical support:

*I have a matron pass so that just lets you go to the matron whenever you want . . . and I have a piece of paper in my bag, I have to hand that to the matron and they read that and it’s got instructions on what to do. (Noah)*

Marie was less supported. She explained the strategy she had developed to manage her anxieties about having ready access to top-ups:

*Not being able to eat when I need it was a bit worrying because I was thinking something could happen in that space of time I wasn’t allowed to eat or drink. It got easier because I learnt I can try and go to the toilet if I need that drink or something to eat, or just wait until the next break. It got a bit easier in secondary school with time because I have to manage it. (Marie)*

The need for top-ups commonly meant dispensations from school regulations about eating and drinking which increased the visibility of MCADD among peers. Several had felt pressured to explain their condition in response to questions from classmates. Frankie *“had to tell them”* when his classmates in primary school *“said why are you eating in class*” and Noah was similarly asked why he was *“allowed to drink Lucozade instead of water.”*

The majority wanted to maintain control over who knew about their MCADD and made a clear distinction between those inside and outside their friendship circle. They all felt it was beneficial for their friends to know about their condition, because of the protection and support it offered. Noah suggested *“it would be weird”* if they did not and Nadia explained it was important *“just in case if something bad happens.”* Mia similarly highlighted the value of her friends who have *“always got my back . . .like if I’m ever ill or anything they’ll always stay with me.”* By contrast, wider information sharing was problematic. The majority were frustrated by the implicit expectation from peers that they justify why they were having top-ups. For two of the older girls, the situation was more worrying. Persistent questioning and challenge about their condition from classmates was experienced as bullying and causing emotional distress.

*“It’s always like oh how come you can eat and we can’t and you can bring sugary drinks in to school and we’re not allowed it and things like that.” (Mia)*

*The other week. . . these girls were making fun of the fact that I’ve got MCADD and they were saying it’s not real and I’ve actually got diabetes and they’re saying it’s not overly serious but it could be. And it put me in a really bad place. I was really down because it really affected me. (Marie)*

### Theme 2. Growing into a Self-Management Role

This theme explores two aspects of self-management. Firstly, the young people’s overall awareness and understanding of their condition and how that had developed over time, and secondly how they were assuming increasing responsibility for managing their condition.

## Understanding my Condition

All the young people demonstrated some understanding of the dietary management requirements of their condition. Simon, Sarah, Mia, Noah, and Amber identified the safe fasting interval of 12 hours and the importance of not exceeding that interval. Nadia spoke of the need for frequent meals during the day explaining that *“usually it’s good to eat slightly above four [hours]”* while Alley explained that *“MCADD is you have to eat every two hours.”* The majority spoke of the need to eat “*sugary foods and starch foods*” (Frankie).

Some, including Charmaine highlighted the dilemma this presented for them, in terms of a balanced diet: *“I have to [have chocolates and sweets], I’m not saying they don’t help me, but they’re not a healthy choice but I have to have them because of the sugar.” (Charmaine)*

Most dated their initial awareness of having MCADD to the age of five or six. Some recalled their parents explaining it to them, including Noah whose mother had *“made it sound quite easy.”* Whilst some of the younger participants’ understanding of their condition was limited to basic dietary requirements, a substantial proportion of the young people however, across the age bands, demonstrated a more detailed scientific understanding. Sarah explained the metabolic nature of the condition when she said *“Not eating for MCADD is really bad. . .because we can’t store fat . . . we need carbohydrates so many hours.”* Mia’s explanation offered metabolic and genetic information: *“I understand that I’ve got an enzyme missing in my liver that breaks my fats down to turn into energy.”*

This level of understanding was most commonly attributed to regular explanations by parents and healthcare professionals that reinforced management principles and ongoing nutritional guidance from the specialist dietician which was an integral part of routine clinical reviews. This enabled understanding to develop over time as Marie and Frankie explained;

“I know they [my parents] helped, just explained it every often and in a way that I would understand it at that age. As I got older they’d explain it more technically to me”. (Marie)

It was when I went to see him [the metabolic consultant]. He would tell me about it and keep reminding me to always have my snacks at school and eat my breakfast and supper in the morning and night. (Frankie)

Others had exercised a considerable degree of agency, seeking out online resources including podcasts aimed at young people and NHS websites to further their understanding. A 11 year old Nadia exemplifies this situation;

*When I was eight and seven I just thought mum and dad would know everything about it, and it’s fine, they’ll look after me. But then I started to just take matters into my own hands, and realise what it is when I was about nine. . .. It’s like you’re a bit older, as I started getting more sick I just realised I needed to do, I started doing more research and all that. I think it’s really helped and paid off. (Nadia)*

## Working Toward Independent Management

All the young people reported a high level of parental supervision over their diet. It involved scrutinizing food labels to check for coconut which is prohibited for those with MCADD because as Jasmin explained *“it will make me a bit ill.”* Sarah’s mum *“checks food before she buys it for coconut, and* [for school meals] *literally, like I need a menu printed out for your school.”*

It also included finding foods with the requisite balance of carbohydrates and sugars. Mia identified the challenges this presented for her and her mum: *“That’s hard to shop for as well, because it’s got to be low sugar but high carbs for slow release. Reading packet, that’s hard when we go to the supermarket.”*

Parents also checked intake at meals, particularly breakfast, and included regular reminders to ensure they kept their energy levels topped up. Frankie explained that *“mum reminds me sometimes that I need to have sugary thing so I don’t be sick”* and 15 year old Sarah reported *“My mum and dad are like, Sarah, you need to eat. My dad’s like, you need to come and get a snack.”*

Several described the strategies they had developed with their parents to support self-management. A 11 year old Yasmin’s mum had provided her with a calibrated beaker and was encouraging her to learn how to make up SOS, the emergency sugar drink that is used for periods of illness. Mia had established a routine so she never slept beyond 11 hours to protect the safe fasting interval. Nadia had embraced the idea, devised with her mum, of using her mobile phone to keep a food diary to help her monitor her own dietary intake:

*I just document it in a way just to give me a little helping hand so I know when I eat when there’s an important time, because if I don’t then I might get sick or something, because I’m really low on energy. (Nadia)*

Many of the youngest participants had little to say about the prospect of adulthood and those that did viewed it with equanimity. Amber saw it in terms of *“making sure I get myself to the doctors and appointments”* and Charmaine was confident she would have been well prepared by parents and healthcare providers *“so I think I’d know the right amount to take my own responsibility.”* Noah who held a similar viewpoint also highlighted the problems of speculating so far ahead: *“It’s probably just all responsibility in my own hands. So I’d probably just have like SOS in a cupboard or something. But it’s quite hard to think of when you’re a young age isn’t it.”*

The older participants, for whom adulthood was a closer reality, offered a more varied and detailed viewpoint. Several reported that their parents were worried about the effects of alcohol, however they indicated it was not a particular concern for them. Two of the older girls were concerned about possible impact on career choices and a wider area of concern was the long-term responsibility for staying safe and well. Mia explained that the prospect of *“having to watch what I’m doing and eating and things like that . . . having to think about it all the time and going out and things like that, that worries me a lot.”* In contrast, Marie was confident in her ability to self-manage in adulthood *“because it has got easier to handle as I’ve grown older.”*

A more imminent issue for some was the transfer from pediatric to adult healthcare services for all their healthcare needs. They were anxious about getting to know and work with new metabolic specialists but a greater concern for some, especially Mia, was the larger scale of adult services and the potential need to access adult emergency services and independently advocate for her health needs within that setting. Those concerns stemmed from previous experience of emergency admissions which were characterized by staff with minimal knowledge of MCADD who had allowed her condition to deteriorate by failing to provide rapid treatment despite advance warning the condition.

*When I go to A&E they leave me there for hours. . .I just don’t understand why if they [the ambulance service] ring up and they [the emergency department] say they’re ready for us, why can’t they just be there and just get me on a drip and then I’ll be fine . . . I just don’t know (Mia)*

## Discussion

This study provides the first insights into the reality of living with MCADD from birth. All the participants understood the basic management principles of MCADD which they articulated in terms of regular meals to protect the safe fasting interval and frequent snacks to keep the energy levels topped up. Even the youngest had acquired some of the key skills of self-management and self-monitoring skills required to adhere to those management principles whilst the older participants were clearly also making decisions about dietary choices which reflected some understanding of the complexity of nutritional balance. In relation to other conditions where food choices are a fundamental aspect of control and management, our study participants had acquired these self-management skills at a comparable age to young people with celiac disease (Fishman et al., 2018) and at an earlier age than those with type 1 diabetes, and food allergies (Markowitz et al., 2015; Monks et al., 2010). This early acquisition had enabled the young people to successfully navigate the transition from primary to secondary education and offers long term benefits in terms of dealing with the challenges associated with increasing social independence. The rarity of MCADD creates a sense of vulnerability in parents of young children who consequently adopt a highly risk averse approach to managing their child’s condition (Piercy et al., 2017; Timmermans & Buchbinder, 2013). Our findings offer some insights into the value of their long-term therapeutic relationships with the metabolic specialist healthcare team of physician, nurse, and dietician who actively supported the development of self-management skills from early childhood so that as their child grew older, parental approaches had translated into continued close supervision and surveillance. The ongoing emotional care support provided by specialist metabolic nurses plays a key role in this and may be particularly important in those cases where there are additional emotional or psychosocial needs.

Schatz and Ensenauer (2010) suggest that one of the risks associated with MCADD is that adolescents and young adults may undervalue the potential consequences of their condition and forget about the need for preventive measures. Studies involving food allergies suggest these types of concerns are well founded. Monks et al. (2010) reported that a high proportion of the 11 to 18 years olds in their study did not adhere to allergy avoidance requirements or carry an EpiPen for use in emergency situations and similar findings were reported by Greenhawt et al. (2009). However, our findings suggest a different reality for MCADD. The young people were acutely aware of the potential consequences of their condition and this gave rise to their main areas of anxiety. They were concerned about the need to maintain adequate energy input and were fearful if they did not have ready access to the means by which to do so, because of the potentially dire consequences. There was no indication that any of the young people had undervalued the risks and “tested” the limits of their condition or that they had become complacent about the need to act preventively by maintaining energy intake. The feeling of running out of energy that many described served to safeguard them from any complacency in this respect because it served as a frequent somatic reminder that they had to act on.

The eating requirements needed to keep them safe created a burden of responsibility for the young people, which was particularly evident among the older participants, for whom it was compounded by the feeling that they had to be always eating. As well as being more independent and autonomous and consequently having a greater sense of self-reliance, the older participants were at a more advanced stage of puberty. Absolute basal metabolic rate and total daily energy expenditure are higher in pubertal adolescents as compared to pre-pubertal adolescents (Cheng et al., 2016) increasing the food requirements. This resulted in a heavy reliance on the use of frequent snacks to provide energy tops-up and that sense of having to be continually eating which effectively characterized their condition. Specialist nutritional advice and guidance helped them respond to and accommodate these changing metabolic demands and should continue to be available after the transfer from pediatric to adult services at age 16 years to ensure long term health benefits.

We had no sense that food security was an issue for participants and their families, however it is likely to affect some families affected by MCADD. Population level data demonstrates that growing up in a food insecure household is associated with poor child health outcomes at population level and food security screening leading to referrals for support are recommended (Drennen et al., 2019). Food insecurity would have a disproportionate impact on those living with MCADD and should be considered in those families identified at risk. The long-term relationships that metabolic nurses establish with families means they are well placed to identify and respond appropriately to concerns of food insecurity.

Other studies have reported specific parental concerns about the potential consequences of adolescent behaviors, notably alcohol consumption and altered sleep patterns for those with MCADD (Torkelson & Trahms, 2010). These concerns were also evident in our study, but only insofar as they represented the parental perspective. The study participants were not yet of an age where these behaviors were a major concern. The younger ones had not considered them at all and the older ones, for whom they were a more imminent reality had adopted a pragmatic position to managing the risk. Whilst encouraging, the extent to which they will maintain these attitudes and behaviors as they move closer toward older adolescence and early adulthood is clearly unknown. A review of the evidence suggests that whilst young people with chronic conditions may not be substantially more likely to engage in risky behavior than their peers, they are doubly disadvantaged because of the increased potential for adverse health outcomes of those behaviors (Sawyer et al., 2007). Continued follow up will help to establish the extent to which this becomes an issue of concern and what input and support is required to reduce the likelihood of adverse outcomes.

It is critically important that those with MCADD effectively transition to adult healthcare services in view of the rarity of the condition and the possible consequences of metabolic crisis. Our study participants are well placed for effective transition which requires that the process starts early and focuses on encouraging independence in the young person without undermining parental involvement (van Staa et al., 2011). In many countries, specialist adult services for rare metabolic conditions are limited and underdeveloped (Suddaby et al., 2020; Trefz et al., 2015). This is a matter of concern given that continued access to a specialist metabolic multidisciplinary team, particularly in late adolescence and early adulthood during the early transition years is likely to influence long term health outcomes in this population.

## Strengths and Limitations

There are a number of strengths and limitations to this study. Our approach to recruitment enabled us to achieve an adequate sample, which can be particularly challenging for projects involving rare conditions. The high response rate means that the socio-demographic profile of the participants will reflect that of the clinically determined population. However, we did not specifically collect demographic details which would have enabled us to report on the extent to which those from minority or marginalized groups contributed to the study. The 6-year age range of the participants provided some longitudinal perspective and is another strength of the study although the variability it provides means that we cannot claim to have achieved data saturation. Finally, the study was conducted in a single site and the specialist care and support which participants receive from this regional specialist metabolic service will have shaped their experiences. The extent to which this is available elsewhere is unknown and this may therefore limit the applicability of the findings.

## Conclusion

Living with MCADD creates specific challenges for young people as they progress through the early phase of adolescence. Early acquisition of self-management skills and ongoing support of parents and the multidisciplinary metabolic healthcare team play a key role in enabling them to deal with those challenges.
